# The Utility of Social Media Recruitment to Achieve a More Diverse Participant Sample

**DOI:** 10.1093/geroni/igab046.3168

**Published:** 2021-12-17

**Authors:** Faith-Christina Washington, Tai-Te Su, Aileen Griffin, Jacob Sosnoff, Shannon Meija

**Affiliations:** 1 University of Illinois at Urbana-Champaign, Champaign, Illinois, United States; 2 University of Illinois at Urbana-Champaign, United States; 3 University of Illinois at Urbana-Champaign, Aurora, Illinois, United States; 4 School of Health Professions, University of Kansas Medical Center, Kansas City, Kansas, United States; 5 University of Illinois, Champaign, Illinois, United States

## Abstract

The COVID-19 pandemic created an immediate, lasting impact on recruitment methods in academic research, most notably in the field of gerontology. To protect older adult participants' health during the COVID-19 crisis, the Daily Balance Project, a 30-day micro-longitudinal study of older adults' awareness of balance in daily life, shifted to complete remote administration. Our new remote protocol included developing new methodologies to recruit participants with varying degrees of fall risk and educational attainment. In this study, we present our approach to remote online recruitment and compare educational attainment, objective and subjective fall risk, and alignment of objective/subjective fall risk across three samples recruited via a) Fall Clinic registry (16 participants); b) University e-newsletter to faculty and staff (5 participants); c) social media recruitment (7 participants). Eligibility included being 65+ and wireless internet at home. For samples a and b, screening assessments were conducted via phone while baseline assessments were conducted in-person. For sample c, screener and baseline assessment were virtual. Analysis of recruitment methods aims to determine whether recruitment via social media platforms may provide a sample of participants with more variation in fall risk or alignment of subjective versus objective balance. Results demonstrate no significant differences in educational attainment (p=0.7949) or balance confidence (p=0.213), despite significant differences in the alignment of objective and subjective fall risk (p=0.031). Participants from samples a and b proved more able to accurately assess fall risk, while sample c had the most misalignment between subjective and objective fall risk assessments.

